# Diagnostic Approach for the Differentiation of the Pandemic Influenza A(H1N1)v Virus from Recent Human Influenza Viruses by Real-Time PCR

**DOI:** 10.1371/journal.pone.0009966

**Published:** 2010-04-01

**Authors:** Martin Schulze, Andreas Nitsche, Brunhilde Schweiger, Barbara Biere

**Affiliations:** 1 Robert Koch-Institut, Fachgebiet 17 Influenza/Respiratorische Viren, Berlin, Germany; 2 Robert Koch-Institut, Zentrum für Biologische Sicherheit 1, Berlin, Germany; Institute of Molecular and Cell Biology, Singapore

## Abstract

**Background:**

The current spread of pandemic influenza A(H1N1)v virus necessitates an intensified surveillance of influenza virus infections worldwide. So far, in many laboratories routine diagnostics were limited to generic influenza virus detection only. To provide interested laboratories with real-time PCR assays for type and subtype identification, we present a bundle of PCR assays with which any human influenza A and B virus can be easily identified, including assays for the detection of the pandemic A(H1N1)v virus.

**Principal Findings:**

The assays show optimal performance characteristics in their validation on plasmids containing the respective assay target sequences. All assays have furthermore been applied to several thousand clinical samples since 2007 (assays for seasonal influenza) and April 2009 (pandemic influenza assays), respectively, and showed excellent results also on clinical material.

**Conclusions:**

We consider the presented assays to be well suited for the detection and subtyping of circulating influenza viruses.

## Introduction

Influenza A and B viruses of the *orthomyxoviridae* family are a cause of significant disease burden in the human population [1]–[3]. In April 2009, a new variant of an influenza A(H1N1) virus (A(H1N1)v) was identified that had never been isolated before, but spread efficiently in the human population [4]. The sudden emergence of this new virus not only demanded a rapid development of specific diagnostic tools, but also challenged a diagnostic routine that in many laboratories was focussed on subtype-independent virus detection only. To fully assess the epidemiological situation, a differentiation of influenza A subtypes is necessary, especially while A(H1N1)v circulation coincides that of seasonal influenzaviruses. Also, subtype identification can influence decisions regarding antiviral therapy, because the different subtypes have a differing antiviral resistance profile [5]–[8]. Thus, a fast determination of the influenza A subtype can directly contribute to patient management. We therefore present a bundle of real-time PCR assays which enable a fast and precise determination of virus type and subtype in respiratory specimens, including the new influenza A(H1N1)v virus. These assays have been used successfully by the German National Reference Center for Influenza since 2007 and April 2009, respectively.

## Results

Real-time PCR assays for the detection and subtyping of relevant human influenzaviruses were designed in consideration of the high variability of these viruses, i.e. their most stable regions were chosen as primer and probe targets. An assay for the subtype-independent detection of influenza A viruses is supplemented with a set of PCR systems that allow for a differentiation of their hemagglutinin and neuraminidase subtypes including the newly emerging swine influenza A(H1N1)v virus. Additionally, an oligonucleotide set for the detection of influenza B viruses was designed (all primer and probe sequences listed in Table 1).

**Table 1:** Primer and probe sequences.

Assay	primer	oligonucleotide sequence (5′–3′)	nM	segment	detected viruses
AMsw	M+25	AgATgAgTCTTCTAACCgAggTCg	300	M	Influenza A, generic
	M-124BB	ccWgCAAARACATCYTCAAgTYTCTg	1200		
	M-124sw	CTgCAAAgACACTTTCCAgTCTCTg	100		
	M+64 MGB	FAM - TCAggCCCCCTCAA - MGB	100		
BM	BMP-13	gAgACACAATTgCCTACCTgC	300	M	Influenza B, generic
	BMP-102AN	TTCCCACCRAACCARCARTgTAAT	1200		
	BMP-72 MGB	FAM - CTgCTTTgCCTTCTC - MGB	100		
H1	FluA H1 F832	ggATCAggAATCATCAMYTCAAATgC	300	HA	seasonal Influenza A(H1N1)
	FluA H1 R959	ggACACTCTCCTATTgTgACTgggTg	300		
	FluA H1 MGB914	FAM - CTgCTgTTTATAgCTCC - MGB	100		
H3	H3F-162	TCCTCATCAgATCCTTgATg	300	HA	seasonal Influenza A(H3N2)
	H3R-291	ACAgTTgCTgTAggCTTTgC	300		
	H3S-284 MGB	FAM - CTCTATTgggRgACCC - MGB	100		
H1v	FluSw H1 F236	TgggAAATCCAgAgTgTgAATCACT	300	HA	pandemic Influenza A(H1N1)v
	FluSw H1 R318	CgTTCCATTgTCTgAACTAgRTgTT	300		
	FluSw H1 TM292	FAM - CCACAATgTAggACCATgAgCTTgCTgT - BHQ1	100		
N1	N1P-1078 AN	AYggYAATggTgTYTggATMggRAg	1200	NA	seasonal Influenza A(H1N1)
	N1P-160 bp	ARCTYCCRCTRTAYCCHgACCARTCRgT	1200		
	N1S MGB	FAM - TCCAYCCRTTRggRTCCCAAA - MGB	100		
N2	N2P-P-769 AN	gATACTAAAATACTATTCATTgAggAgg	300	NA	seasonal Influenza A(H3N2)
	N2P 934 AN	ATATCTACDATgggCCTATTggAgC	300		
	N2-S-840 MGB	FAM - CAYTCCTCgACATgCTg - MGB	100		
N1v	FluSw N1 F1255	AgACCTTgCTTYTgggTTgAAC	300	NA	pandemic Influenza A(H1N1)v
	FluSw N1 R1334	AAggATATgCTgCTCCCRCTAgT	300		
	FluSw N1 TM1310	FAM - CAgATTgTgTTCTCTTTgggTCgCCCT - BHQ1	100		
FCV	FVC F54	CGTTACCGCCACACCCAT	300	n.a.	Feline Calicivirus
	FCV R141	GAGTTCACGAAAGATTTCAGACCAT	300		
	FCV TM96	TexasRed - ACCCATCATTCTAACACTCCCGCCAAT - BHQ2	100		

All oligonucleotide sequences are listed in 5′–3′ orientation. M = Matrix, HA = Hemagglutinin, NA = Neuraminidase, n.a. = not applicable.

For each PCR assay, a plasmid was produced that contains the respective PCR target sequence. In case of the generic influenza A assay (AM), plasmids were produced for different virus subtypes that currently circulate in the human population (H1N1, H3N2, H5N1, H1N1v) to allow for a judgement on the amplification of all relevant influenzaviruses.

For all assays, a linear dynamic range of 10^6^ to 10^1^ genome equivalents was found. Due to three oligonucleotide mismatches in the reverse primer our original AM assay amplified the newly emerging pandemic H1N1v virus with a reduced analytical sensitivity as compared to the influenza A subtypes H1N1, H3N2 and H5N1 (data not shown). Therefore, a third primer (M-124 sw) was added to the oligonucleotide mixture in May 2009 to compensate for these mismatches (AMsw assay). This modification of the original AM assay increased the performance for H1N1v significantly while preserving the performance characteristics for the other subtypes (all validation data summarized in Table 2). The AMsw assay shows stable amplification efficiency and a high correlation of the standard curve for the different subtypes tested. The limit of detection (95% detection probability) was determined to be around six genome equivalents per reaction. The intra- as well as interassay variability (standard deviation of Ct values) was shown to be ≤0.81 even for low copy numbers. The AMsw assay can be duplexed with the FCV assay (Feline Calicivirus, sample internal control) without a significant loss of performance, as only the detection limit is minimally raised, while efficiency and variability of AMsw remain unaltered.

**Table 2:** PCR assay validation results.

Assay^a^	(sub)type	Slope^b^	E^b^	R^2^ b	LOD^c^	Reproducibility^d^					
						Intrassay			Interassay		
						500,000	5,000	50	500,000	5,000	50
AMsw	A(H1N1)	−3.18	106%	0.999	5.95	0.29	0.22	0.58	0.36	0.33	0.81
	A(H3N2)	−3.42	96%	0.998	4.99	0.13	0.12	0.35	0.24	0.22	0.48
	A(H1N1)v	−3.32	100%	0.999	7.11	0.09	0.13	0.34	0.35	0.25	0.46
	A(H5N1)	−3.68	87%	0.999	6.79	0.27	0.14	0.46	0.39	0.18	0.40
AMsw+FCV	A(H1N1)	−3.55	91%	0.999	12.81	0.19	0.13	0.59	0.20	0.44	0.61
H1	A(H1N1)	−3.40	97%	0.998	2.81	0.10	0.11	0.21	0.14	0.21	0.36
H3	A(H3N2)	−3.70	86%	0.997	4.92	0.14	0.21	0.46	0.25	0.29	0.54
H1v *	A(H1N1)v	−3.14	108%	0.987	14.33	0.12	0.27	0.67	0.22	0.29	0.52
N1	A(H1N1)	−3.39	97%	0.998	6.49	0.18	0.16	0.51	0.31	0.16	0.41
N2	A(H3N2)	−3.46	95%	0.999	6.79	0.18	0.10	0.28	0.16	0.09	0.54
N1v *	A(H1N1)v	−3.30	101%	0.999	9.76	0.32	0.21	0.55	0.29	0.32	0.64
BM	B	−3.27	102%	0.999	5.61	0.14	0.22	0.73	0.19	0.22	0.78

a validation data was obtained on a Stratagene Mx3000 instrument for the assays indicated with an asterisk. All other assays were validated on a Stratagene Mx3005 instrument.

b slope, efficiency (E) and correlation (R^2^) of standard curve; PCR efficiency was calculated as E = 10^(−1/slope)^−1.

c Limit of detection (LOD) was calculated as 95% detection probability by probit analyses applying the SPSS 17.0 Statistics software.

d reproducibility was calculated by examination of indicated plasmid copy numbers; intraassay: sixfold examination in a single run, interassay: twofold examination of double reactions plus inclusion of intraassay data; standard deviation of obtained Ct values are listed.

Similar performance characteristics were found for the PCR assays for subtype determination. The H1, H3, N1 and N2 assays all have a good PCR efficiency with a high standard curve correlation and a low assay imprecision. The 95% detection probability was found to be between 2.81 and 6.79 genome equivalents per reaction.

The newly developed assays for the detection of H1N1v hemagglutinin and neuraminidase genes also allow a sensitive detection of this new virus subtype. The 95% detection probability was shown to be 14.33 (H1v) and 9.76 (N1v) genome equivalents per reaction, with also a high standard curve correlation and a low assay variability.

Also for the influenza B virus assay such performance characteristics were found. The PCR efficiency approached 100%, again with a high standard curve correlation, low assay variability and a 95% detection probability of approximately six genome equivalents per reaction.

Cross-reactivities were tested with all relevant influenza types and subtypes (22× pandemic A(H1N1)v, 12× seasonal A(H1N1), 12× A(H3N2), 6× Influenza B Yamagata lineage, 6× Influenza B Victoria lineage) as well as 6 A(H5N1) isolates, 6 A(H2N2) isolates, 6 A(H7) isolates and 3 A(H9) isolates. Additionally, isolates of Human respiratory syncytial virus A and B, Human metapneumovirus, Human parainfluenzavirus 1–4, Human adenovirus types 2–4, Human coxsackievirus A6 and B1, Human enterovirus 71, Human echovirus 9, 11 and 30, Human poliovirus 1–3, Chlamydia pneumoniae and Mycoplasma pneumoniae were tested. Furthermore, approximately 20 human virus-negative swab materials were examined for cross-reactivities to human genomic DNA. No cross-reactivities were observed for either assay with non-targeted pathogens or human genomic DNA.

**Figure 1:**
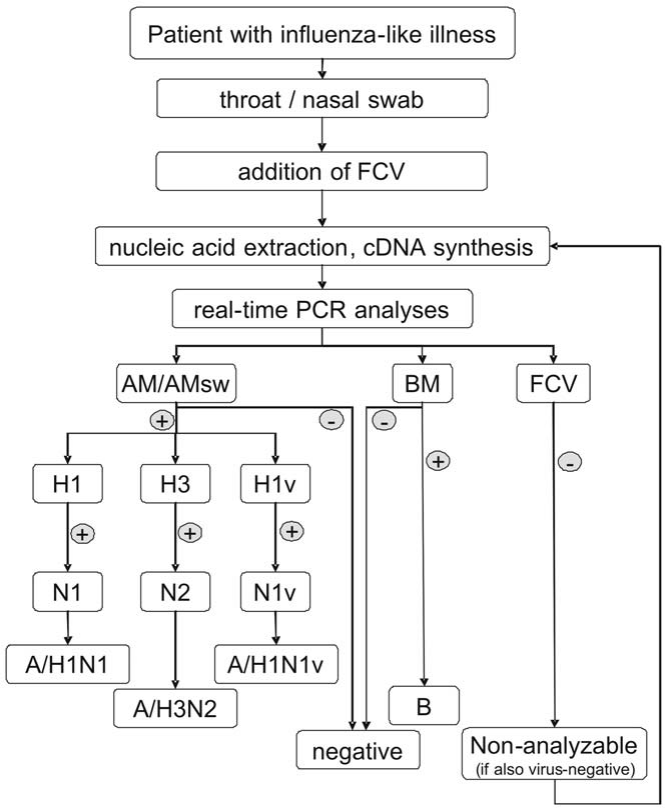
Real-time PCR diagnostic scheme. Samples are spiked with FCV after arrival in the lab. After nucleic acid extraction and cDNA synthesis, all samples are examined by real-time PCR with the AM/AMsw + FCV duplex PCR assay and the BM assay. All influenza A-positive samples then undergo subtyping by the H1, H3 and H1v assays. The subtype information can be completed by the application of the corresponding N1, N2 and N1v assays. FCV-negative samples are considered non-analyzable in case that no influenza A or B virus is amplified, and are re-analyzed. Otherwise, samples are considered positive for the detected virus.

All assays for the detection of seasonal influenza viruses were validated on approximately 80–100 primary samples containing the corresponding types and subtypes. These samples had been diagnosed by other PCR assays and furthermore in part had been confirmed by hemagglutination inhibition testing (HIT) after virus culture. These validations did not indicate any problems concerning their analytical sensitivity, their cross-reactivity or their detection capability of viruses in the field. Therefore, the assays (with the exception of AMsw and A(H1N1)v assays) were transferred to our diagnostic routine procedure in 2007 (Figure 1): Before nucleic acid extraction, FCV is added to the sample material to yield approximately 50 genome equivalents per PCR template volume of cDNA. All samples then are examined with the AM/AMsw, BM and FCV assays. In case of a negative FCV result in an influenzavirus-negative sample, the sample gets re-extracted and/or a new cDNA is synthesized. In influenza A positive samples, the hemagglutinin and neuraminidase subtypes are identified.

Since 2007, the assays for seasonal influenza viruses have been run on approximately 1000 (N1, N2), 4000 (H1, H3) or 6000 (BM) primary samples. Until the emergence of A(H1N1)v viruses in April 2009 also the AM assay had been run on approximately 15000 samples, while the enhanced AMsw assays since then has been run on approximately 4000 samples. For all assays, the examination of primary material yielded results that were in concordance with additional or complementary PCR assays (e.g. assays for subtype identification) regarding Ct values and overall results. Also, all results matched those from antigenic characterization (hemagglutination inhibition testing) of virus isolates after successful virus culture. Influenza A-positive samples could always be typed as A(H1N1), A(H3N2) or A(H1N1)v, and to our knowledge we never received any false-positive results.

The validation of the H1v assay was performed on clinical samples in parallel with two additional real-time PCRs (specific for A(H1N1)v and swine influenza viruses, respectively) as well as two conventional (nested) PCR assays that were furthermore confirmed by sequencing of the amplicon (Sanger sequencing or Pyrosequencing, respectively). We continued to closely monitor the possible occurrence of false-positive or false-negative results for approximately the first 100 samples. By then, our validation on H1N1v-negative material had shown that cross-reactivities to other pathogens or human genomic DNA did not occur. We continued to confirm H1v-positive samples by at least one of the methods mentioned above for approximately 1000 samples. The N1v assay was introduced into our diagnostic routine after a regular validation on the above mentioned material as well as all available H1v-positive samples within few weeks after the emergence of A(H1N1)v. Since then, it has been run on approximately 3500 samples and always yielded results in congruence to all other PCR data.

## Discussion

After the emergence of the pandemic influenza A(H1N1)v virus in April 2009, PCR assays for the specific detection of this novel virus variant were internationally communicated almost instantly. Many laboratories picked up the national and international recommendations and were able to offer A(H1N1)v PCR diagnostics within few days or weeks. Also, PCR assays were published very quickly [9]–[11], so that diagnostic expertise was distributed further. However, especially during the early phase of pandemic virus circulation, a co-circulation of the seasonal influenza A (and B) viruses has to be expected, and indeed was observed on the southern hemisphere from spring to fall 2009 [12]–[15]. Therefore, precise diagnostics including subtype determination are important for a comprehensive epidemiological surveillance. Also, a subtype identification is relevant for appropriate patient management, because the seasonal and pandemic influenza A viruses cause severe disease in differing patient groups [16], [17] and have a different resistance profile [5]–[8]. Routine diagnostics are often limited to influenza A (and B) virus detection, while virus subtyping is predominantly performed for surveillance purposes and thus mainly restricted to laboratories with public health duties. To provide interested laboratories with methods for this task, we present a bundle of PCR assays to be used for subtype identification. These assays have been used by the German National Reference Center for Influenza for the preceding influenza seasons as well as during the emergence of the pandemic A(H1N1)v virus.

The PCR for the generic amplification of influenza A viruses targets the viral M segment and thus allows a virus detection that is independent of the viral subtype. Its oligonucleotide design is based on a primer/probe system described by Spackman et al. [18]. Slight modifications of primer sequences as well as substitution of the conventional 5′-exonuclease probe with a Minor Groove Binder (MGB) probe resulted in an assay that amplifies influenza A subtypes H1N1, H3N2 and H5N1 with similar analytical sensitivity and reproducibility. Unfortunately, its performance on the pandemic H1N1v virus proved to be impaired due to three nucleotide mismatches in the reverse primer. The addition of a third primer to the oligonucleotide mix that perfectly matches the H1N1v sequences yielded a significant improve in assay performance with H1N1v viruses without reducing the performance on the other subtypes H1N1, H3N2 and H5N1.

Also for the detection of seasonal H1 and N1 as well as H3 and N2 subtypes, PCR assays were designed to implement MGB probes. The MGB molecule stabilizes the DNA duplex formation by hydrophobic interactions, which results in an increase in the probes melting temperature and thus allows the design of shorter probes [19]. This not only offers improved options for the detection of highly variable sequences like those from influenzaviruses, but also leads to an increase in fluorescence intensity and sometimes a shift of Ct values of several cycles when compared to conventional probes (unpublished data). These features are especially helpful for samples containing only few copies of viral genome, since not only the detection limit is improved, but also the interpretation of dubious results is simplified. We therefore prefer MGB probes to conventional 5′-exonuclease probes in real-time PCR diagnostics. On the other hand, MGB probes are more vulnerable to nucleotide mismatches compared to conventional probes [19], and therefore a close monitoring of the genetic virus evolution is imperative to ensure the suitability of the MGB assays in the future. For the same reason we also decided to apply conventional probes for the detection of pandemic A(H1N1) viruses, because in April 2009 the assay designs had to be based on single sequences without any knowledge concerning the viruses sequence variability. The application of MGB probes might have possibly produced dubious or false negative results in a fraction of samples in case of variable nucleotides within the MGB's target sequence. For these first-generation assays we therefore consider a conventional probe to be more suitable because of their higher tolerance to nucleotide mismatches, but surely these assays will have to be updated or completely changed with increasing knowledge of viral sequences over time [20], including the design of MGB probes if possible. So far, the high degree of viral sequence homology has not yet necessitated these assay updates, but surely the A(H1N1)v viruses will start to evolve like the seasonal influenza viruses when they are confronted with an increasingly immune population.

After an extensive validation of the developed assays with plasmid dilutions as well as defined primary sample material, each newly developed assay was transferred to our diagnostic routine which includes the examination of all samples with the AM/AMsw, BM, and the FCV assays. The latter ensures a sample check for inhibitory substances and preparation failures which otherwise could result in false-negative diagnostic findings [21]. To further simplify this procedure, the FCV assay was combined with the AM assay to a duplex PCR (AM: FAM reporter, FCV: TexasRed reporter). This duplex approach did not result in a deterioration of PCR performance, as all relevant characteristics remained unaltered. This finding holds also true for the modified AMsw assay, as was demonstrated by our validation efforts.

In our lab, all influenza A-positive samples are further characterized regarding their hemagglutinin subtype by the application of the seasonal H1 and H3 assays. Additionally, the corresponding N1 or N2 assay is run on a representative subset (during peak phase of influenzavirus circulation) or even all samples. To further simplify the diagnostic procedure we combined the H1 and H3 as well as the N1 and N2 assays to duplex PCRs. Preliminary data indicates that this duplexing does not lead to relevant assay impairment, but yields results that are comparable to the singleplex performance.

All assay validation data were generated by using dilutions of plasmids containing the respective assay target sequences. We consider this kind of validation to be preferable to viral cDNA preparations because of its higher objectivity and thus comparability to assay performances observed in other laboratories. The preparation of viral cDNAs is greatly influenced by lab-specific preparation protocols regarding RNA/cDNA quality and quantity, and initial virus particle quantity can only be roughly estimated by classical virological means like hemagglutination inhibition testing or virus titration. Also, we found the validation data obtained with plasmid dilutions to be very similar to data obtained with dilutions of influenzavirus material [22], so that we do expect this to hold true in general.

During the first days of the emergence of the pandemic A(H1N1)v virus the described assays were used to (i) verify an infection with an influenza A virus and (ii) exclude seasonal influenza A subtypes. Within only four days after publication of the first viral sequences, the H1v assay was designed, validated and distributed to the federal German health authorities and laboratories. Only little time later, the N1v assay could be included into the diagnostic routine. Both the H1v and the N1v assay were designed not only to detect, but also to discriminate A(H1N1)v from the seasonal A(H1N1) (and A(H3N2)) viruses. Indeed, no cross-reactivity was observed even for virus isolate preparations with a very high virus load.

In total, we consider the presented assays to be valuable tools for influenza virus typing and subtyping. The plasmid validation data indicate sensitive and reproducible virus detection, while the assays performance on thousands of clinical samples ensures their applicability in clinical diagnostics. We therefore believe that our set of PCR assays will greatly contribute to virus detection and subtyping during and after the H1N1v pandemic.

## Methods

### Clinical samples

The clinical samples were taken from patients presenting with influenza-like illness who gave verbal consent for virological examination. Samples were sent to the National Reference Center for Influenza at the Robert Koch Institute (Berlin, Germany) for influenzavirus surveillance purposes in Germany. Consequently, ethic committee approval was not required since such a sentinel surveillance is covered by German legislation (Infektionsschutzgesetz §§13, 14). Since 2007, approximately 16000 nasal and/or throat swabs (and other materials like throat and nasal washings, bronchoalveolar lavages etc.) have been collected.

### Sample preparation, nucleic acid extraction and cDNA synthesis

Swabs were washed out in cell culture medium either individually or pooled per patient. RNA was extracted from 300 µL culture supernatant using the MagAttract Viral RNA M48 Kit (Qiagen) and eluted in 80 µL elution buffer. Alternatively, RNA was extracted using the RTP DNA/RNA Virus Mini Kit (Invitek) from 400 µL culture supernatant with an elution volume of 60 µL. 25 µL of extracted RNA were subjected to cDNA synthesis applying 200 U M-MLV Reverse Transcriptase (Invitrogen) in a total reaction volume of 40 µL.

### Assay Design

Sequence alignments for the selection of PCR target regions were generated using the BioEdit 7.0.9 software after sequence download from NCBI (National Center for Biotechnology Information, http://www.ncbi.nlm.nih.gov). Primer and probe oligonucleotide sequences were designed using the Oligo 6.71 and Primer Express 3.0 software.

### Polymerase Chain Reaction

Quantitative real-time PCR was carried out on Mx3000 and Mx3005 real-time PCR thermal cyclers (Stratagene) in a total reaction volume of 25 µL. The reaction contained 1× PCR buffer, 5 mmol/L MgCl_2_, 1 mmol/L dNTP (Invitrogen) with dUTP (GE Healthcare), 0.5 U Platinum Taq Polymerase (Invitrogen), primers (Metabion, Tib Molbiol) and probes (Applied Biosystems, Metabion, Tib Molbiol) in differing concentrations (listed in Table 1), and 3 µL of cDNA. After 5 minutes at 95°C for Taq DNA polymerase activation, a total of 45 cycles consisting of denaturation at 95°C for 15 seconds and annealing at 60°C for 30 seconds were performed. After the run, data were analyzed using the software MxPro.

### Plasmids

Fresh PCR products were cloned using the TOPO TA Cloning Kit (Invitrogen) according to the manufacturer's instructions. Plasmid DNA was extracted from 1 mL of bacterial culture using the Invisorb Spin Plasmid Mini Two Kit (Invitek) as recommended by the manufacturer. For verification of correct sequences at the primer and probe binding sites, plasmids were sequenced using the dye terminator chemistry (ABI-Prism Big Dye Terminators v3.1 Cycle Sequencing Kit, Applied Biosystems) in a 3130xl Genetic Analyzer (Applied Biosystems). The obtained sequences were analyzed, assembled and aligned using the DNA STAR Software Package. Plasmid preparations were diluted in λ–DNA (1 ng/µL) for PCR assay validations.
